# An Application of Maximal Exponential Models to Duality Theory

**DOI:** 10.3390/e20070495

**Published:** 2018-06-27

**Authors:** Marina Santacroce, Paola Siri, Barbara Trivellato

**Affiliations:** Dipartimento di Scienze Matematiche “G.L. Lagrange”, Dipartimento di Eccellenza 2018-2022, Politecnico di Torino, Corso Duca degli Abruzzi 24, 10129 Torino, Italy

**Keywords:** maximal exponential model, Orlicz spaces, convex duality, exponential utility

## Abstract

We use maximal exponential models to characterize a suitable polar cone in a mathematical convex optimization framework. A financial application of this result is provided, leading to a duality minimax theorem related to portfolio exponential utility maximization.

## 1. Introduction

In this manuscript, we use the notion of maximal exponential model in a convex duality framework and consider an application to portfolio optimization problems.

The theory of non-parametric maximal exponential models centered at a given positive density *p* starts with the work by Pistone and Sempi [1]. In that paper, by using the Orlicz space associated with an exponentially growing Young function, the set of positive densities is endowed with a structure of exponential Banach manifold.

More recently, different authors have generalized this structure replacing the exponential function with deformed exponentials (see, e.g., Vigelis and Cavalcante [2], De Andrade et al. [3]) and in other directions (see e.g., Imparato and Trivellato [4]).

The geometry of nonparametric exponential models and its analytical properties in the topology of Orlicz spaces have been also studied in subsequent works, such as Cena and Pistone [5] and Santacroce, Siri and Trivellato [6,7], among others.

Statistical exponential models built on Orlicz spaces have been exploited in several fields, such as differential geometry, algebraic statistics, information theory and physics (see, for example, Brigo and Pistone [8], Lods and Pistone [9]).

In mathematical finance, convex duality is strongly used to tackle portfolio optimization problems. Very recently, exponential models have been exploited for the first time to address the classical exponential utility maximization problem (see Santacroce, Siri and Trivellato [7]).

In this paper, we consider a particular set of densities contained in the maximal exponential model centered at a fixed density *p*, and we give a characterization of its polar cone with respect to a suitable dual system, studied in Santacroce, Siri and Trivellato [10].

This characterization is then exploited to slightly improve a well known financial result on exponential utility maximization. In particular, we prove that the classical optimal strategy, which solves the primal problem, maximizes the expected utility over a larger set.

The paper is organized as follows. In Section 2, we recall Orlicz spaces and related properties from the literature and some results on maximal exponential models, contained in our previous works and in papers by Pistone and different coauthors. Section 3 contains the main results of the paper, namely the characterization of the polar cone associated with the set of densities belonging to the maximal exponential model and under which a given convex set of random variables has negative expectation. In Section 4, a financial application of this characterization is given and leads to a minimax theorem for exponential utility maximization.

## 2. Orlicz Spaces and Maximal Exponential Models

In this section, we recall some results from the theory of Orlicz spaces and exponential models that we will use hereinafter.

We denote with Φ a *Young function*, which is an even, convex function from R to [0,+∞] such that

(i) Φ(0)=0,
(ii) $\lim_{x\to \infty }\Phi \left(x\right)=+\infty$,
(iii) Φ(x)<+∞ in a neighborhood of 0.

Two Young functions Φ and $\Phi^{\prime }$ are said to be *equivalent* if there exists $x_{0}>0$, and $0<c_{1}<c_{2}$ such that $\Phi \left(c_{1}x\right)\leq \Phi^{\prime }\left(x\right)\leq \Phi \left(c_{2}x\right)$, for any $x\geq x_{0}$.

In the following, we will focalize on $\Phi_{1}\left(x\right)=\cosh\left(x\right)-1$, which is equivalent to the more commonly used $\Phi_{2}\left(x\right)=e^{\left|x\right|}-\left|x\right|-1$.

Let us recall that the *conjugate convex function* $F^{*}$ of a real function *F* is defined as

(1) $$F^{*}\left(y\right)=\underset{x\in R}{\sup}\left\{xy-F\left(x\right)\right\},\;\forall y\in R,$$

and that the Fenchel inequality

(2) $$xy\leq F\left(x\right)+F^{*}\left(y\right),\;\forall x,y\in R$$

holds. In the case of a Young function Φ the conjugate, denoted by Ψ, is itself a Young function and the stronger Fenchel–Young inequality

(3) |xy|≤Φ(x)+Ψ(y),∀x,y∈R

holds.

The conjugate of the Young function $\Phi_{1}$ is $\Psi_{1}\left(y\right)=\int_{0}^{y}\sinh^{-1}\left(t\right)dt$ and is equivalent to the conjugate of $\Phi_{2}$, namely $\Psi_{2}\left(y\right)=\left(1+|y|)\log(1+|y|)-|y\right|$.

From now on, we will work on a fixed probability space (X,F,μ). We denote with P the set of all densities that are positive μ-a.s. and with $E_{p}$ the expectation with respect to pdμ, for each fixed p∈P.

Given p∈P, we consider the Orlicz space associated with a Young function Φ, defined by

(4) $$L^{\Phi }\left(p\right)=\left\{u:X\to R\;\;\mathrm{measurable}:\exists \;\alpha >0\;s.t.\;E_{p}\left(\Phi \left(\alpha u\right)\right)<+\infty \right\}.$$

$L^{\Phi }\left(p\right)$ is a Banach space when endowed with the *Luxembourg norm*

(5) $${∥u∥}_{\Phi ,p}=\inf\left\{k>0\;:\;E_{p}\left(\Phi \left(\frac{u}{k}\right)\right)\leq 1\right\}.$$

The Orlicz spaces $L^{\Phi }\left(p\right)$ and $L^{\Phi^{\prime }}\left(p\right)$ associated with two equivalent Young functions are equal as sets and have equivalent norms as Banach spaces.

Finally, it is worth noting the following chain of inclusions:

$$L^{\infty }\left(p\right)\subseteq L^{\Phi_{1}}\left(p\right)\subseteq L^{a}\left(p\right)\subseteq L^{\psi_{1}}\left(p\right)\subseteq L^{1}\left(p\right),\;a>1.$$

**Definition** **1.** *p,q∈P are connected by an open exponential arc if there exists an open interval I⊃[0,1] such that the following equivalent relations are satisfied:*

*1.*  *$p\left(\theta \right)\propto p^{\left(1-\theta \right)}q^{\theta }\in P$, ∀θ∈I;*
*2.*  *$p\left(\theta \right)\propto e^{\theta u}p\in P$, ∀θ∈I, where $u\in L^{\Phi_{1}}\left(p\right)$ and p(0)=p,p(1)=q.*

Observe that connection by open exponential arcs is an equivalence relation.

**Definition** **2.** *Let us denote $L_{0}^{\Phi_{1}}\left(p\right)=\left\{u\in L^{\Phi_{1}}\left(p\right):E_{p}\left(u\right)=0\right\}.$ The cumulant generating functional is the map $K_{p}:L_{0}^{\Phi_{1}}\left(p\right)⟶\left[0,+\infty \right]$ defined by the relation $K_{p}\left(u\right)=\log E_{p}\left(e^{u}\right)$.*

We recall from Pistone and Sempi [1] that $K_{p}$ is a positive convex and lower semicontinuous function, vanishing at zero. Moreover, the interior of its proper domain

(6) $$\mathrm{dom}\;K_{p}=\left\{u\in L_{0}^{\Phi_{1}}\left(p\right):K_{p}\left(u\right)<+\infty \right\},$$

denoted here by $\overset{\circ }{\mathrm{dom}\;K_{p}}$, is a non-empty convex set.

**Definition** **3.** *For every density p∈P, the maximal exponential model at p is defined as*

(7) $$E\left(p\right)=\left\{q=e^{u-K_{p}\left(u\right)}p:u\in \overset{\circ }{dom\;K_{p}}\right\}\subseteq P.$$

In the sequel, we use the notation D(q∥p) to indicate the Kullback–Leibler divergence of Q=q·μ with respect to P=p·μ and we simply refer to it as the divergence of *q* from *p*.

**Proposition** **1.** *Let p,q∈P, then $D\left(q∥p\right)<+\infty \;⟺\;\frac{q}{p}\in L^{\Psi_{1}}\left(p\right)\;⟺\;\log\frac{q}{p}\in L^{1}\left(q\right)$.*

**Proposition** **2.** *Let p,q∈P. If D(q∥p)<+∞, then $L^{\Phi_{1}}\left(p\right)\subseteq L^{1}\left(q\right)$.*

The proof of Proposition 1 can be found in Cena and Pistone [5], while Proposition 2 is proved in Santacroce, Siri and Trivellato [6].

We now state one of the central results of [5,6,7], which gives equivalent conditions to open exponential connection by arcs, in a complete version, containing all the recent improvements.

**Theorem** **1.** *(Portmanteau Theorem)*

*Let p,q∈P. The following statements are equivalent.*

*(i)*  *q∈E(p);*
*(ii)*  *q is connected to p by an open exponential arc;*
*(iii)*  *E(p)=E(q);*
*(iv)*  $\log\frac{q}{p}\in L^{\Phi_{1}}\left(p\right)\cap L^{\Phi_{1}}\left(q\right);$
*(v)*  *$L^{\Phi_{1}}\left(p\right)=L^{\Phi_{1}}\left(q\right)$;*
*(vi)*  *$\frac{q}{p}\in L^{1+\varepsilon }\left(p\right)$ and $\frac{p}{q}\in L^{1+\varepsilon }\left(q\right),$ for some ε>0;*
*(vii)*  *the mixture transport mapping*
  (8) $$\begin{array}{cc} {}^{m}U_{p}^{q}:L^{\Psi_{1}}\left(p\right) & ⟶L^{\Psi_{1}}\left(q\right)\\ v & \mapsto \frac{p}{q}v, \end{array}$$
  *is an isomorphism of Banach spaces.*

The equivalence of conditions (i)÷(iv) is proved in Cena and Pistone [5]. Statements (v) and (vi) have been added by Santacroce, Siri and Trivellato [6], while statement (vii) by Santacroce, Siri and Trivellato [7]. It is worth noting that, among all conditions of the Portmanteau Theorem, (v) and (vi) are the most useful from a practical point of view: the first one allows for switching from one Orlicz space to the other at one’s convenience, while the second one permits working with Lebesgue spaces. On the other hand condition (vii), involving the mixture transport mapping could be a useful tool in physics applications of exponential models, as the recent research on the subject demonstrates (see, e.g., Pistone [11], Lods and Pistone [9], Brigo and Pistone [8]). In these applications, finiteness of Kullback–Leibler divergence, implied from Portmanteau Theorem, is a desirable property.

**Corollary** **1.** *If q∈E(p), then the Kullback–Leibler divergences D(q∥p) and D(p∥q) are both finite.*

The converse of this corollary does not hold, as the counterexamples in Santacroce, Siri and Trivellato [6,7] show.

**Proposition** **3.** *Let p∈P. Then, E(p) is a convex set.*

For the proof, see Santacroce, Siri and Trivellato [6].

## 3. Duality Results

This section contains the main results of the paper, namely it shows an application of the maximal model to duality in a general framework.

We start with introducing as basic tools $K\subseteq L^{0}\left(p\right)$ a convex cone containing 0 and

(9) $$M\left(K\right)=\{q\in P_{\geq }\;:\;-\infty <E_{q}\left(k\right)\leq 0,\;\;\forall \;k\in K\},$$

where $P_{\geq }$ denotes the set of non negative densities. Let us define

(10) $$M_{E}\left(K\right)=M\left(K\right)\cap E\left(p\right)$$

and suppose $M_{E}\left(K\right)\neq \emptyset$.

Furthermore, let us introduce the linear spaces

(11) $$L=\underset{q\in M_{E}\left(K\right)}{\cap }L^{1}\left(q\right)⊇K\;\;\;\mathrm{and}\;\;\;L^{\prime }=Lin\left\{M_{E}\left(K\right)\right\}\subseteq L^{1}\left(p\right).$$

*L* and $L^{\prime }$ are dual spaces with the duality given by the bilinear map $\left(l,l^{\prime }\right)\to \langle l,l^{\prime }\rangle =E_{\mu }\left(ll^{\prime }\right).$

The dual system is separated in both *L* and $L^{\prime }$ and, endowed with the weak topologies, they become locally convex Hausdorff topological vector spaces (see Santacroce, Siri and Trivellato [10] for details on this duality).

In the following, we denote with $M_{E}^{0}\left(K\right)$ the polar cone of $M_{E}\left(K\right)$, i.e.,

(12) $$M_{E}^{0}\left(K\right)=\left\{h\in L\;|\;E_{q}\left(h\right)\leq 0\;\;\forall q\in M_{E}\left(K\right)\right\}.$$

The following result gives a characterization of the density that minimizes the divergence over $M_{E}\left(K\right)$ in a formulation that is very close to the one given by Biagini and Frittelli [12]. Therefore, we omit the proof, even if the fact that we work on the exponential model makes some checking more straighforward.

**Proposition** **4.** *$\bar{q}\in M_{E}\left(K\right)$ minimizes D(q∥p) over $M_{E}\left(K\right)$ if and only if $D\left(\bar{q}∥p\right)-\log\frac{\bar{q}}{p}\in M_{E}^{0}\left(K\right)$.*

In the next theorem, we give the main result of the paper, namely a useful description of the polar cone in terms of K.

**Theorem** **2.** (13) $$M_{E}^{0}\left(K\right)=\underset{q\in M_{E}\left(K\right)}{\cap }{\bar{K-L_{+}^{1}\left(q\right)}}^{q},$$

*where ${\bar{C}}^{q}$ represents the $L^{1}\left(q\right)$-closure of a set C.*

**Proof.**  The technique used in the proof is in the spirit of that in Biagini and Frittelli [12]. On the other hand, here we need to work with measures belonging to E(p). This adds a difficulty to the proof which can be solved by exploiting the properties of maximal exponential models given by the Portmanteau Theorem (i.e., Theorem 1).

We prove only that

$$M_{E}^{0}\left(K\right)\subseteq \underset{q\in M_{E}\left(K\right)}{\cap }{\bar{K-L_{+}^{1}\left(q\right)}}^{q},$$

since the opposite inclusion essentially follows from the definition of $M_{E}\left(K\right)$.

Let $h\in M_{E}^{0}\left(K\right)$ and suppose by contradiction that there exists $q_{0}\in M_{E}\left(K\right)$ such that $h\notin {\bar{K-L_{+}^{1}\left(q_{0}\right)}}^{q_{0}}$.

By the Hahn–Banach Theorem, there exists $\eta \in L^{\infty }\left(q_{0}\right)$ such that for every $k\in K-L_{+}^{1}\left(q_{0}\right)$

(14) $$E_{q_{0}}\left[\eta k\right]\leq 0<E_{q_{0}}\left[\eta h\right].$$

If we consider $k=-1\;\;1_{\left(\eta <0\right)}$, by (14), we get $E_{q_{0}}\left[\eta 1\;\;1_{\left(\eta <0\right)}\right]=0$ and thus η≥0 μ-*a.s.*.

Then, we can define a new density $q_{1}=\frac{\eta }{E_{q_{0}}\left[\eta \right]}q_{0}$ (μ-*a.s.*). Let us note that $q_{1}\geq 0$ does not belong to P but, by (14), $q_{1}\in M\left(K\right)$ and, $E_{q_{1}}\left[h\right]>0$.

Now consider the convex combination $q_{\lambda }=\lambda q_{1}+\left(1-\lambda \right)q_{0}$, with 0≤λ<1.

We can prove that $q_{\lambda }\in M_{E}\left(K\right)$, for any 0≤λ<1. It is clear that $q_{\lambda }\in M\left(K\right)\cap P$. In order to check that it belongs to the maximal exponential model, we use statement vi) of Theorem 1. In fact,

(15) $$\begin{array}{c} E_{p}\left[{\left(\frac{q_{\lambda }}{p}\right)}^{1+\epsilon }\right]=E_{p}\left[{\left(\lambda \frac{\eta }{E_{q_{0}}\left[\eta \right]}\frac{q_{0}}{p}+\left(1-\lambda \right)\frac{q_{0}}{p}\right)}^{1+\epsilon }\right]\leq C\;E_{p}\left[{\left(\frac{q_{0}}{p}\right)}^{1+\epsilon }\right],\; \end{array}$$

(16) $$\begin{array}{c} E_{p}\left[{\left(\frac{p}{q_{\lambda }}\right)}^{\epsilon }\right]=E_{p}\left[\frac{1}{{\left(\lambda \frac{\eta }{E_{q_{0}}\left[\eta \right]}+1-\lambda \right)}^{\epsilon }}{\left(\frac{p}{q_{0}}\right)}^{\epsilon }\right]\leq C\;E_{p}\left[{\left(\frac{p}{q_{0}}\right)}^{\epsilon }\right], \end{array}$$

where the inequalities are due to the fact that η is non negative and bounded.

Since $q_{0}\in E\left(p\right)$, by vi) of Theorem 1, we get $E_{p}\left[{\left(\frac{q_{0}}{p}\right)}^{1+\epsilon }\right]<+\infty$ and $E_{p}\left[{\left(\frac{p}{q_{0}}\right)}^{\epsilon }\right]<+\infty$. Moreover, from (15) and (16), we deduce $E_{p}\left[{\left(\frac{q_{\lambda }}{p}\right)}^{1+\epsilon }\right]<+\infty$ and $E_{p}\left[{\left(\frac{p}{q_{\lambda }}\right)}^{\epsilon }\right]<+\infty$. Therefore, $q_{\lambda }\in E\left(p\right)$ and consequently $q_{\lambda }\in M_{E}\left(K\right)$.

Finally, let us denote by $\beta =E_{q_{1}}\left[h\right]>0$ and $\alpha =E_{q_{0}}\left[h\right]\leq 0$, since $q_{0}\in M_{E}\left(K\right)$ and $h\in M_{E}^{0}\left(K\right)$. It is easy to see that $E_{q_{\lambda }}\left[h\right]=\lambda \beta +\left(1-\lambda \right)\alpha >0$ if and only if $\lambda >\frac{-\alpha }{\beta -\alpha }$.

By definition of the polar cone, we get to a contradiction and this concludes the proof. ☐

## 4. Financial Application

In this section, we endow the probability space $\left(X,F,\mu \right)$ with a filtration $F={\left(F_{t}\right)}_{0\leq t\leq T}$ satisfying the usual conditions, and $F=F_{T}$, where T∈(0,∞] is a fixed time horizon. We fix p∈P and consider P=∫pdμ. Let $X={\left(X\right)}_{0\leq t\leq T}$ be a real-valued continuous semimartingale, which represents the discounted price of a risky asset in a financial market.

We denote by M the set of all probability densities $q=\frac{dQ}{d\mu }$, where Q is a P-absolutely continuous *local martingale measure* for *X*, which is a probability measure absolutely continuous with respect to P such that *X* is a local $\left(F,Q\right)$-martingale. Without the risk of misunderstanding, when saying that *X* is a *q*-local martingale, with q∈M, we will intend that *X* is a local martingale with respect to Q. Moreover, let $M^{e}$ be the subset of M consisting of those densities *q* that are strictly positive μ-a.s. and define

(17) $$M_{f}=\left\{q\in M\;:\;D\left(q∥p\right)<\infty \right\},\;M_{f}^{e}=M_{f}\cap M^{e}.$$

We assume throughout the paper that $M_{f}^{e}\neq \emptyset$.

Note that, by Corollary 1,

$$M\cap E\left(p\right)=M_{f}\cap E\left(p\right)=M_{f}^{e}\cap E\left(p\right).$$

A *self-financing trading strategy* is denoted by $\theta ={\left(\theta_{t}\right)}_{0\leq t\leq T}$, where $\theta_{t}$ represents the number of shares invested in the asset. We assume that θ is in L(X), that is an F-predictable and *X*-integrable process. The stochastic integral process W(θ)=θ·X=∫θdX is then well defined and, assuming an initial capital equals to zero, $W_{t}\left(\theta \right)$ represents the portfolio wealth at time *t*.

Let $U\left(x\right)=-e^{-\gamma x}$ be the exponential utility function with risk aversion parameter γ∈(0,+∞) (without loss of generality we will set γ=1). Consider the related problem of maximizing the expected utility of the final wealth

(18) $$\underset{\theta \in \Theta }{\sup}E_{p}\left[U(W_{T}\left(\theta \right))\right]$$

over a set Θ of *admissible strategies*.

In this framework, Θ is the classical set of strategies where wealth is uniformly bounded from below (see for example Schachermayer [13]):

(19) $$\Theta =\{\theta \in L\left(X\right):W_{t}\left(\theta \right)\geq c\;\mu -a.s.,\;\forall \;0\leq t\leq T,\mathrm{for}\;\mathrm{some}c\in R\}.$$

It is well known that the maximization problem in (18) can be written in the duality formulation

(20) $$\underset{\theta \in \Theta }{\sup}E_{p}\left[U(W_{T}\left(\theta \right))\right]=U\left(\underset{q\in M_{f}}{\inf}D\left(q∥p\right)\right).$$

Recall that, if $M_{f}\neq \emptyset$, then there exists a unique $q^{*}\in M_{f}$ that minimizes D(q∥p) over all $q\in M_{f}$ (see Theorem 2.1 in Frittelli [14]). This $q^{*}$ is called the *minimal entropy martingale (density) measure*. If, in addition, $M_{f}^{e}\neq \emptyset$, then $q^{*}>0$ μ-a.s.

Let q∈P and denote the two densities projections $q_{t}=E_{\mu }\left(q|F_{t}\right)$ and $p_{t}=E_{\mu }\left(p|F_{t}\right)$.

**Definition** **4.** *$\left(R_{L\log L}\left(p\right)\right)$ We say that q satisfies the Logarithmic Reverse Hölder inequality with respect to p, if there exists a constant C>0 such that*

(21) $$E_{p}\left[\frac{q/p}{q_{\tau }/p_{\tau }}\log\left(\frac{q/p}{q_{\tau }/p_{\tau }}\right)|F_{\tau }\right]\leq C\;\;\;for\;all\;stopping\;times\;\;\tau \leq T.$$

If there exists $q\in M_{f}^{e}$ which satisfies $R_{L\log L}\left(p\right)$, then the minimal entropy martingale measure $q^{*}$ also satisfies $R_{L\log L}\left(p\right)$ (see e.g., Lemma 3.1 in Delbaen et al. [15]). When the process *X* is continuous, this fact then implies that $q^{*}\in E\left(p\right)$, as the following proposition shows.

**Proposition** **5.** Let X be a continuous semimartingale and assume there exists $q\in M_{f}^{e}$ that satisfies $R_{L\log L}\left(p\right)$. Then, $q^{*}\in E\left(p\right)$.

(For the proof, see Santacroce, Siri and Trivellato [7]).

Let us consider here the convex cone whose elements are the final values of the portfolio wealth

(22) $$K=\{W_{T}\left(\theta \right):\theta \in \Theta \}.$$

The maximization problem can then be rewritten in terms of K:

(23) $$\underset{k\in K}{\sup}E_{p}\left[U(k)\right].$$

Moreover, M(K) in (9) coincides with the set of martingale measures M. For the sake of coherence, we drop the dependence on K also in $M_{E}\left(K\right)$ in (10) and $M_{E}^{0}\left(K\right)$ in (12), denoting them respectively $M_{E}$ and $M_{E}^{0}$.

It is common knowledge that the optimal solution to the primal utility maximization problem (23) on the class K does not exist, but it exists on the larger set $K_{f}=\underset{q\in M_{f}}{\cap }{\bar{K-L_{+}^{1}\left(q\right)}}^{q}$ (see, e.g., Biagini and Frittelli [12]).

The following theorem, exploiting the results in the previous section, proves that this set can be further enlarged if the minimal martingale measure $q^{*}$ belongs to E(p).

**Theorem** **3.** *Let X be a continuous semimartingale and assume there exists $q\in M_{f}^{e}$ which satisfies $R_{L\;log\;L}\left(p\right)$. Then,*

(24) $$\underset{k\in K}{\sup}E_{p}\left(U(k)\right)=\max_{k\in K_{E}}E_{p}\left(U(k)\right)=U\left(\min_{q\in M_{E}}D\left(q∥p\right)\right)=U\left(\min_{q\in M_{f}}D\left(q∥p\right)\right),$$

*where*

(25) $$K_{E}=\underset{q\in M_{E}}{\cap }{\bar{K-L_{+}^{1}\left(q\right)}}^{q}.$$

**Proof.**  It is trivial to note that $K_{f}\subseteq K_{E}$ so that $\underset{k\in K_{f}}{\sup}E_{p}\left(U(k)\right)\leq \underset{k\in K_{E}}{\sup}E_{p}\left(U(k)\right)$.

Using the duality results from Biagini and Frittelli [12], we get

$$\underset{k\in K}{\sup}E_{p}\left(U(k)\right)=\underset{k\in K_{f}}{\sup}E_{p}\left(U(k)\right)=\max_{k\in K_{f}}E_{p}\left(U(k)\right)=U\left(\min_{q\in M_{f}}D\left(q∥p\right)\right).$$

On the other side, using Fenchel inequality (2) and exploiting Theorem 2, we get

$$\underset{k\in K_{E}}{\sup}E_{p}\left(U(k)\right)\leq U\left(\underset{q\in M_{E}}{\inf}D\left(q∥p\right)\right).$$

In fact, for any $k\in K_{E}=M_{E}^{0}$ and $q\in M_{E}$, by definition of polar cone

$$E_{p}\left(U(k)\right)\leq E_{p}\left(ke^{-D(q∥p)}\frac{q}{p}\right)+E_{p}\left(V\left(e^{-D(q∥p)}\frac{q}{p}\right)\right)\leq E_{p}\left(V\left(e^{-D(q∥p)}\frac{q}{p}\right)\right),$$

where V(x)=xlog(x)−x is the convex conjugate of the exponential function.

Since $E_{p}\left(V\left(e^{-D(q∥p)}\frac{q}{p}\right)\right)=-e^{-D(q∥p)}=U\left(D(q∥p)\right)$, we get

$$\underset{k\in K_{E}}{\sup}E_{p}\left(U(k)\right)\leq \underset{q\in M_{E}}{\inf}U\left(D(q∥p)\right)=U\left(\underset{q\in M_{E}}{\inf}D\left(q∥p\right)\right).$$

Finally, by Proposition 5, $q^{*}\in E\left(p\right)$, from which we deduce that

$$U\left(\underset{q\in M_{E}}{\inf}D\left(q∥p\right)\right)=U\left(\min_{q\in M_{E}}D\left(q∥p\right)\right)=U\left(\min_{q\in M{}_{f}}D\left(q∥p\right)\right)$$

and this concludes the proof. ☐

## 5. Conclusions

We studied maximal exponential models and their use in a mathematical convex optimization framework.

The main result characterizes the polar cone associated with the set of densities belonging to the maximal exponential model and under which a given convex set of random variables has non-positive expectation.

In a financial context, this set describes a “good” set of claims, which is both economically sound and well suited for exploiting convex duality techniques.

As an application we considered an exponential utility maximization problem in a continuous semimartingale incomplete market model and, under some technical assumptions, we proved a minimax theorem.

It turns out that the solution of the primal problem, which cannot be attained in the set of replicable claims by admissible strategies at zero initial cost, is reached on this new larger set of claims described by means of the maximal exponential model.

Further investigation in which the Kullback–Leibler divergence is replaced by other statistical divergences, such as Bregman’s (see e.g., [16,17]), could be done. However, this interesting research would require a suitable theory with powerful tools like the Portmanteau Theorem.

Moreover, the dual results obtained in the present paper could probably be extended in the framework described by [18,19], where a portfolio optimization problem, which involves deformed exponentials, is investigated.
